# Forecasting Institutional LINAC Utilization in Response to Varying Workload

**DOI:** 10.1177/15330338221123108

**Published:** 2022-10-26

**Authors:** Srinivas Raman, Fan Jia, Zhihui Liu, Julie Wenz, Michael Carter, Colleen Dickie, Fei-Fei Liu, Daniel Letourneau

**Affiliations:** 1 Department of Radiation Oncology, 7938 University of Toronto, Toronto, ON, Canada; 2 Radiation Medicine Program, 10051 Princess Margaret Cancer Centre, Toronto, ON, Canada; 3 Department of Industrial Engineering, 7938 University of Toronto, Toronto, ON, Canada; 4 Department of Biostatistics, 10051 Princess Margaret Cancer Centre, Toronto, ON, Canada; 5 Department of Medical Biophysics, 7938 University of Toronto, Toronto, ON, Canada

**Keywords:** LINAC utilization, LINAC forecasting, predictive modeling

## Abstract

**Objectives**Pandemics, natural disasters, and other unforeseen circumstances can cause short-term variation in radiotherapy utilization. In this study, we aim to develop a model to forecast linear accelerator (LINAC) utilization during periods of varying workloads. **Methods:** Using computed tomography (CT)-simulation data and the rate of new LINAC appointment bookings in the preceding week as input parameters, a multiple linear regression model to forecast LINAC utilization over a 15-working day horizon was developed and tested on institutional data. **Results:** Future LINAC utilization was estimated in our training dataset with a forecasting error of 3.3%, 5.9%, and 7.2% on days 5, 10, and 15, respectively. The model identified significant variations (≥5% absolute differences) in LINAC utilization with an accuracy of 69%, 62%, and 60% on days 5, 10, and 15, respectively. The results were similar in the validation dataset with forecasting errors of 3.4%, 5.3%, and 6.2% and accuracy of 67%, 60%, and 58% on days 5, 10, and 15, respectively. These results compared favorably to moving average and exponential smoothing forecasting techniques. **Conclusions:** The developed linear regression model was able to accurately forecast future LINAC utilization based on LINAC booking rate and CT simulation data, and has been incorporated into our institutional dashboard for broad distribution. **Advances in knowledge:** Our proposed linear regression model is a practical and intuitive approach to forecasting short-term LINAC utilization, which can be used for resource planning and allocation during periods with varying LINAC workloads.

## Introduction

Institutional radiotherapy (RT) utilization is generally not subject to significant weekly or monthly fluctuations, allowing adequate time for equipment and workforce capacity adjustment. However, as we describe below, the COVID-19 pandemic caused significant disruptions to our departmental operations, highlighting the need for a robust forecasting model to predict and guide near-term linear accelerator (LINAC) utilization (LU).^1^ During the peak of the pandemic, several guidelines from professional societies were published to advise on RT utilization for cancer patients.^2,3^ The recommendations could be categorized under 2 scenarios: an early pandemic scenario, where the guiding principle was to mitigate exposure risks to patients and staff, and a late pandemic scenario, where severely reduced RT resources could prevent treatment from being delivered to patients in need.^4^

In anticipation of a late pandemic scenario, our department assembled a multidisciplinary task force to ensure there was always an adequate workforce to facilitate RT delivery for all cancer patients. Staffing across all disciplines was tracked daily, and in parallel, LU was monitored as well. One of the challenges identified was that in addition to matching current RT demand to workforce supply, there was a need to estimate these variables up to 3 weeks later, as new patients seen in consultation currently and planned for RT may be impacted at that later time. We had no direct way to estimate the impact of change in new patient consultations, treatment deferrals, altered fractionation regimens, or variation in cancer presentation and treatment intents on LU. Additionally, in a large department such as ours with >8000 new cancer patients seen annually, it was critically important that all staff were aware of the current and future LU to guide critical decision making including whether to defer nonessential treatments if necessary.

A review of the operations research literature was performed, prior to developing an LU forecasting tool. The majority of the published work has focused on optimizing patient scheduling processes for a given patient workload.^5^ In terms of forecasting LU, previous studies have focused on modeling patient flow and using simulation models to derive the LU.^6–8^ In this study, we describe a simple model-based approach that can guide the ramp-up and ramp-down of RT delivery in periods of varying workloads.

## Methods

The study design was a retrospective simulation using LINAC and computed tomography (CT)-simulator booking data from our institution. The booking workflow for our department is that most patients receiving curative treatment are booked for CT simulation first. Once an acceptable RT plan has been created by the dosimetrist, the patient is booked for LINAC appointments. Conversely, most patients receiving palliative or urgent treatment are booked for CT simulation and LINAC appointments at the same time. The proposed method for LU forecasting utilized a multiple linear regression model to predict the daily LU over a 15-working day horizon. The predicted LU, *P_i_* (*i* = days 1-15), was calculated using equation (1) and corresponds to the summation of the appointments already booked on the LINACs (*HB_i_*) for the next 15 days, and the remaining LINAC capacity (model assumes fixed short-term total LINAC capacity) in normal operation, *C_i_* (equation [2]), modulated by an activity-dependent factor *M_i_*. The relative correction factor *M_i_* depends on the average variation in the rate of LINAC appointment booking ($\bar{\Delta B}$) and the average utilization of our CT simulators (*S*) in the last 5 days. Both average LINAC appointment booking rate variation ($\bar{\Delta B}$) and CT-simulator utilization (*S*) were normalized by their corresponding average value during normal operations.

The effect of variations on LINAC appointment booking rate (Δ*B*) and CT-simulator utilization (*S*) was translated into LU in the model using the weighting factors *w_b_* and *w_s_* (equation [3]). These weighting factors were optimized on a daily basis using least squares by minimizing the sum of squared differences between the actual and predicted LU over the last 15 days. Variation in these weighting factors as a function of time could capture changing practice patterns such as altered fractionation, use of specialized RT techniques, and also reflect potential variation in the distribution of cancer types and treatment intents (palliative vs radical) of patients assessed for RT. The LINAC appointment booking rate variation (Δ*B*) and the CT-simulator utilization (*S*) are not independent variables, and day-dependent weighting factors *t_i,b_* and *t_i,s_* (range between 0 and 1, and sum up to 1; Supplemental Appendix Figure A1) were introduced to model the effect that LINAC appointment booking rate may preferentially impact LU in the first 10 days, whereas CT-simulator utilization would likely impact the LU later in the prediction horizon, given that there is a lag between CT-simulator and treatment start dates. Both *t_i,b_* and *t_i,s_* were defined as a 1-dimensional vector (*i* = days 1-15) and their values were changed as part of the *w_b_* and *w_s_* optimization. The LINAC appointment booking rate variation (Δ*B*) was computed using a 5-day difference in booked LINAC hours on days 1 to 15. The average LINAC appointment booking rate variation ($\bar{\Delta B}$) was the mean of booking rate variation from days 1 to 15. The CT-simulator utilization (*S*) was calculated over 5 days, with the use of an exponential smoothing filter to reduce daily variation.

LU, LINAC appointment booking rate, and CT-simulator utilization data were extracted from our radiation oncology information system (Mosaiq, Elekta) from January 1 to September 23, 2020, to develop and test our predictive model. The data was processed to remove duplicate entries and associate each CT simulation appointment with a unique treatment course. The date range was selected to cover 3 months of normal operation prepandemic, and the subsequent 6 months of the pandemic during which significant variations in LU were observed. The performance of the model was assessed by comparing the actual and predicted LU over the entire data period. In addition to testing the numerical differences between the actual and predicted LU, the ability of the model to predict significant variations in LU (>5% absolute change) was investigated. If a >5% change was forecasted in the specified horizon, this was “flagged” as a significant increase or decrease in LU. Confusion matrices were generated to illustrate the recall, precision, and accuracy of the flagged changes. The model was tested in an independent validation cohort, on additional 6 months of data from January to June 2021. The performance of the multiple linear regression model was also compared with the 10-day moving average and simple exponential smoothing forecasting techniques:

(1) $$P_{i}=HB_{i}+M_{i}*C_{i}$$

where

(2) $$C_{i}=F_{\mathrm{normal}}-HB_{i,\mathrm{normal}}$$

(3) $$M_{i}=t_{i,b}*w_{b}*\left(\frac{\bar{\Delta B}}{{\bar{\Delta B}}_{\mathrm{normal}}}\right)+t_{i,s}*w_{s}*\left(\frac{S}{{\bar{S}}_{\mathrm{normal}}}\right)$$

*HB_i_* is the number of hours booked on LINAC on day *i* = 1 to 15 from now. *C_i_* is the remaining hours of LINAC capacity during normal operation. *M_i_* is an activity-based relative correction factor. *HB_i,_*_normal_ is the number of hours booked on LINAC on day *i* = 1 to 15 from now during normal operation. *F*_normal_ is the total average LU during normal operation. *t_i,b_* is a 1-dimensional vector of constant weighting factors for the booking rate ratio for day *i* = 1 to 15. *t_i,s_* is a 1-dimensional vector of constant weighting factors for the CT simulation ratio for day *i* = 1 to 15:

*t_i,b_* + *t_i,s_* = 1

*w_b_* is the optimized weighting factor for the booking ratio on LINAC. *w_s_* is the optimized weighting factor for the CT simulation ratio. $\bar{\Delta B}\;$ is the average 5-day booking rate variation on LINAC from day 1 to 15. ${\bar{\Delta B}}_{\mathrm{normal}}$ is the 5-day average booking rate variation on LINAC during normal operation (days 1 to 15). *S* is the number of CT simulation appointments in the past 5 days before the day of prediction. ${\bar{S}}_{\mathrm{normal}}$ is the average number of CT appointments during normal operations.

## Results

**Figure 1.**
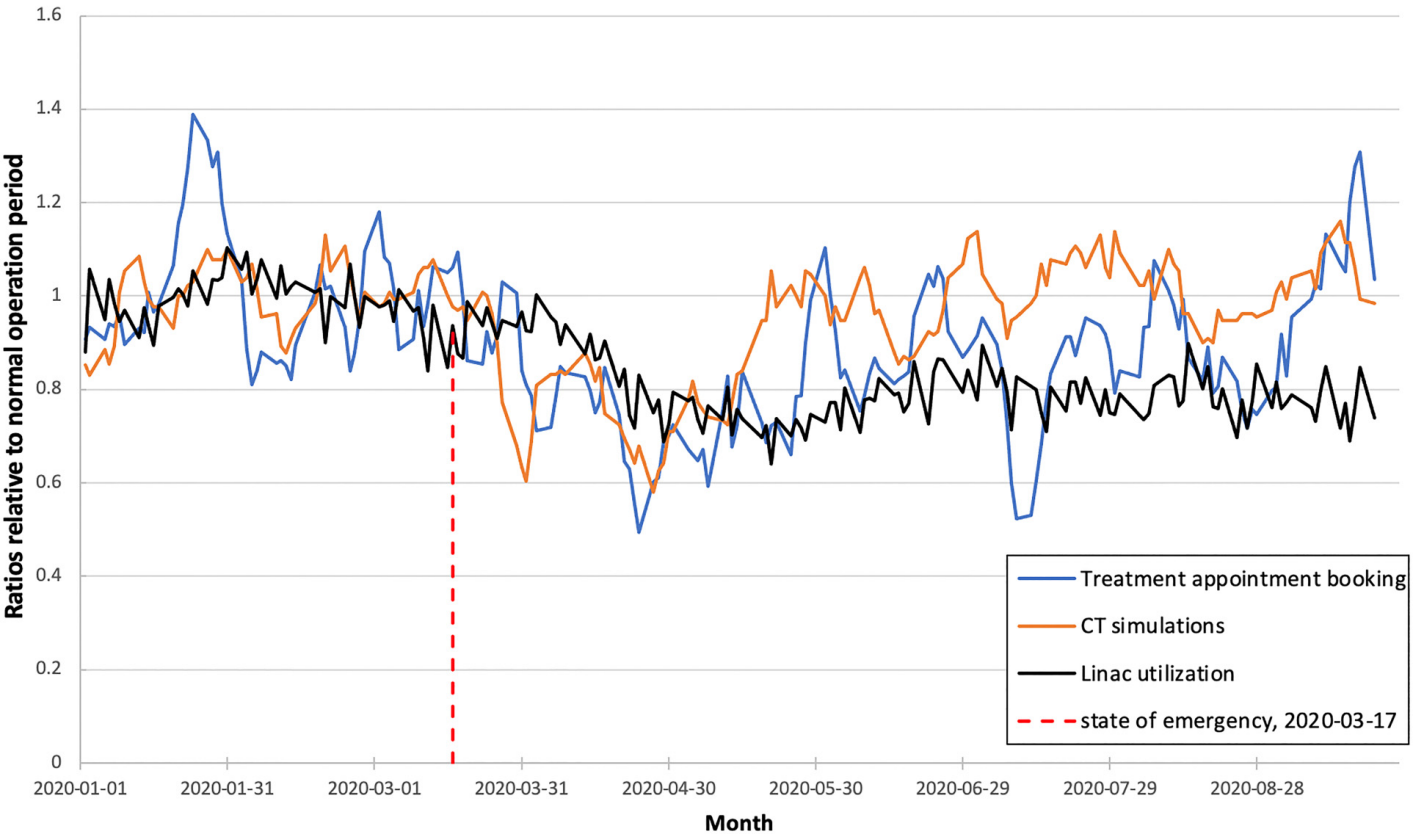
Linear accelerator (LINAC) utilization CT-simulator utilization and booking rate January to September 2020.

Abbreviations: LINAC, linear accelerator; CT, computed tomography.

**Figure 2.**
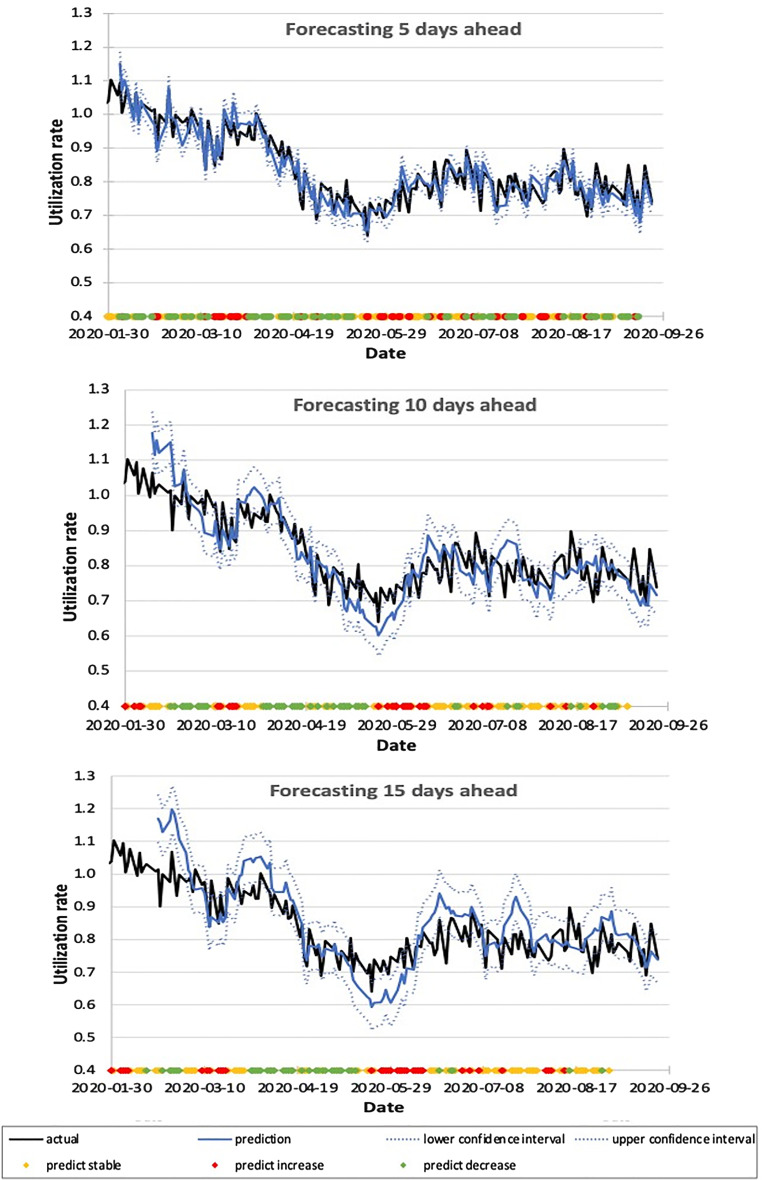
Linear accelerator (LINAC) utilization prediction for days 5, 10, and 15 (3 curves) from January to September 2020. The colored dots represent the predicted changes, where green indicates predicted decrease, red indicates predicted increase, and yellow indicates predicted stability.

In this study, LU was tracked from January 1 to September 23, 2020. In total, 4448 unique courses of LINAC-based RT corresponding to 56,484 fractions were delivered during this period. Supplemental Appendix Figure A2 details how the average number of fractions/treatment course and LINAC time per fraction varied. Overall there was a trend toward hypofractionation with the average number of fractions per new course being 14.4 for January to March 2020 versus 11.9 between April and June 2020 versus 11.6 between July and September 2020. The average LINAC time per fraction did not vary significantly during this time period ranging from 21 to 22 min per appointment. The average waiting time from CT simulation to the first LINAC fraction was 9.0 days. Our institution houses 16 LINACs and 4 CT simulators and data regarding machine utilization was retrieved from the radiation oncology information system. Figure 1 shows how LU and CT simulation and booking rates varied during the study period. An important milestone was March 17, 2020, when a state of emergency was declared in Ontario Canada. This led to the lowest number of appointment bookings, CT simulations, and LU of 50%, 60%, and 70%, respectively, observed in April 2020. Figure 2 shows the performance of the model where daily-predicted LU was compared to the actual LU over a 3-week horizon. Using a definition of 1 standard deviation the prediction intervals for days 5, 10, and 15 LU were 3.3%, 5.9%, and 7.2%, respectively. The performance of the model in predicting changes on days 5, 10, and 15 was evaluated using precision recall and accuracy presented in Table 1. The forecasting performance in the validation cohort is similar to the results during the pandemic with a forecasting error of 3.4%, 5.3%, and 6.2% on days 5, 10, and 15, respectively. The accuracy in this validation dataset was 67%, 60%, and 58% on days 5, 10, and 15. The results of the multiple linear regression model were compared with moving average and exponential smoothing forecasting techniques in Table 2.

**Table 1.** The Performance of Linear Accelerator Utilization (LU) Forecasting for Days 5, 10, 15, and 20 During the Training Period from January to September 2020.

		Recall	Precision	Accuracy
Day 5	Any change (increase or decrease)	0.81	0.61	0.69
	Increase	0.78	0.74	
	Decrease	0.84	0.54	
Day 10	Changes (increase or decrease)	0.65	0.61	0.62
	Increase	0.57	0.53	
	Decrease	0.68	0.64	
Day 15	Changes (increase or decrease)	0.62	0.61	0.60
	Increase	0.63	0.46	
	Decrease	0.57	0.70	

**Table 2.** The Comparison of Different Linear Accelerator Utilization (LU) Forecasting Methods for Days 5, 10, and 15 During the Validation Period from January to June 2021.

		Mean absolute deviation	Accuracy
Day 5	Linear regression model	3.52	0.66
	Moving average	6.01	0.52
	Exponential smoothing	5.59	0.62
Day 10	Linear regression model	5.86	0.61
	Moving average	6.65	0.56
	Exponential smoothing	6.44	0.50
Day 15	Linear regression model	7.41	0.55
	Moving average	7.35	0.54
	Exponential smoothing	7.20	0.50

## Discussion

In response to the institutional need to forecast future LU, we present a simple method to calculate this parameter over a 3-week horizon. Using booking rate and CT simulation data as input parameters a multiple linear regression model was developed. The performance of the model was evaluated and a forecasting error of 3.3%, 5.9%, and 7.2% on days 5, 10, and 15, respectively, were observed. In addition to providing an estimate of future LU, the other utility of this model is to flag any significant changes in order to guide resource planning. The threshold of 5% for a “significant” change was chosen to represent the workload of approximately 1 LINAC, which is staffed by 3 radiation therapists. Until day 15, the performance of the flagging system was reasonable with recall precision and accuracy above 60% for most instances.

The advantage of this model is that it utilizes a generalizable framework for LU forecasting using intuitive parameters such as booking rate and CT simulations. Time-adjusted continuously updating weighted factors account for potential practice changes and variation such as the trend toward hypofractionation as observed in this dataset. There has been other work performed to estimate machine utilization by modeling patient flow in the RT department and healthcare settings.^6^ For example, recently Vieira et al^7^ used discrete-event simulation (DES) to investigate various combinations of push and pull strategies in RT scheduling. The DES model used several inputs including patient arrival rates, processing time, and resource availability.^7^ Similarly Babashov et al^8^ used similar inputs to model patient flow in a large RT department and determine the effect of reduced human resources or increased patient volumes on waiting time targets. Thomas^9^ developed a Monte-Carlo simulation to estimate the LU and determined the spare capacity required to keep patient waiting time low. While simulation models can determine LU by modeling patient flow and determining the number of hours booked on LINACs, the main objectives of the simulation models are to test different scheduling strategies and resource allocation, in order to improve target performance such as reduced patient waiting times. Our forecasting model focuses on using the current CT simulation and LINAC booking rate to estimate the short-term variation of the LU, so the department can plan its resources ahead of time when there is varying patient demand and workload. Further work is now being performed to improve the model performance and prediction horizon using additional input parameters such as radiation consultation information and using more advanced modeling methods. It will be interesting to compare the performance of the proposed linear regression model with more sophisticated modeling techniques such as DES, since the expertise and software for performing more advanced modeling techniques may not be available at all institutions.

One limitation of this work is the need for independent validation on another dataset before robust conclusions can be drawn. The primary challenge with validating this model using separate data is that historically there have been no other time periods in the last 10 years where LU has varied by more than 5% in consecutive weeks; therefore, there is an absence of real-world data to validate this approach. We did test the model on an additional 6 months of data in 2021 when our LU patterns were stabilized and closer to normal operations (Suuplemental Appendix Figure A3) and the forecasting performance was similar to the results during the pandemic with a forecasting error of 3.4%, 5.3%, and 6.2% on days 5, 10, and 15, respectively. In this validation cohort, the performance of the linear regression model compared favorably to the moving average and exponential smoothing forecasting techniques. Specifically, the accuracy of the linear regression model was highest for days 5, 10, and 15 LU forecasts. This supports the inclusion of “predictive” elements with CT simulation data, which may improve forecasting performance during periods of fluctuating/changing workloads given that averaging and smoothing forecasting techniques are based on the assumption that the mean is slowly varying.

We also recognize that size of our center (16 LINACs) and our ability to pool treatment delivery resources greatly reduce the impact of breakdowns on our treatment capacity. However, the forecasting model presented in this work was designed to guide operational strategies related to LU and staffing assignments in situations of both overcapacity and undercapacity. The main utility of this model was during the COVID-19 pandemic when the department was asked to reduce the LU to a target of 60% to 70% during the peak infectious period and ramp-up to full operations after resolution. Access to forecasted LU allowed for a coordinated departmental effort in patient prioritization (to reduce infectious exposure) as well as ensuring adequate infrastructure and staff assignment during the ramping down and up periods. Also, the methods described in this paper to forecast LU are agnostic to institutional capacity and could potentially be scaled to smaller institutions that may have different operational challenges. We recognize that operational characteristics and workflows at other institutions may differ and therefore this framework may need to be adapted for the individual needs of other institutions.

This model only addresses the forecasting component of RT scheduling, and there is ongoing work to develop a schedule optimizer based on the forecasted workload. The approach to schedule optimization is dependent on how incoming patient flow is managed. For example, Thomsen and Nørrevang^10^ presented a tool to optimize capacity in patients with different waiting times. The model assumes a relatively static case mix citing data that the patterns of practice did not significantly change over 1 year. Whereas Crop et al^11^ presented a DES-based Constant-Work-In-Progress model to account for long in-room times high variability and high rate of cancelations. Scheduling models are also used to optimize patient appointment assignments on different LINACs. For example, Saure et al^12^ formulated a Markov decision process model, and Conforti et al^13^ developed a mixed integer programming model to allocate treatment capacity to incoming demand. In these approaches, forecasting machine utilization is not required.

In summary, we have developed a linear regression model to forecast LU which has now been incorporated into clinical dashboards for prospective validation and departmental use. The results of this algorithm can guide human resource planning and LINAC staffing, maintenance scheduling, patient scheduling as well as programmatic planning during periods of varying workload.

## Conclusion

In this study, we present a practical method to forecast future LU based on CT simulation data and the rate of new bookings in the preceding week. This model or variations of this approach could be used for resource planning during periods of varying LINAC workload.

## Supplemental Material

sj-docx-1-tct-10.1177_15330338221123108 - Supplemental material for Forecasting Institutional LINAC Utilization in Response to Varying Workload Click here for additional data file. Supplemental material, sj-docx-1-tct-10.1177_15330338221123108 for Forecasting Institutional LINAC Utilization in Response to Varying Workload by Srinivas Raman, Fan Jia, Zhihui Liu, Julie Wenz, Michael Carter, Colleen Dickie, Fei-Fei Liu and Daniel Letourneau in Technology in Cancer Research & Treatment

## Abbreviations

CT: computed tomography
DES: discrete-event simulation
LINAC: linear accelerator
LU: LINAC utilization
RT: radiotherapy.
