# Supplementary material for: Forecasting Institutional LINAC Utilization in Response to Varying Workload
Source: Technol Cancer Res Treat. 2022 Oct 26;21:15330338221123108. doi: 10.1177/15330338221123108 (PMC9608060; doi:10.1177/15330338221123108)
Supplement: sj-docx-1-tct-10.1177_15330338221123108 - Supplemental material for Forecasting Institutional LINAC Utilization in Response to Varying Workload [file sj-docx-1-tct-10.1177_15330338221123108.docx]

**APPENDIX**


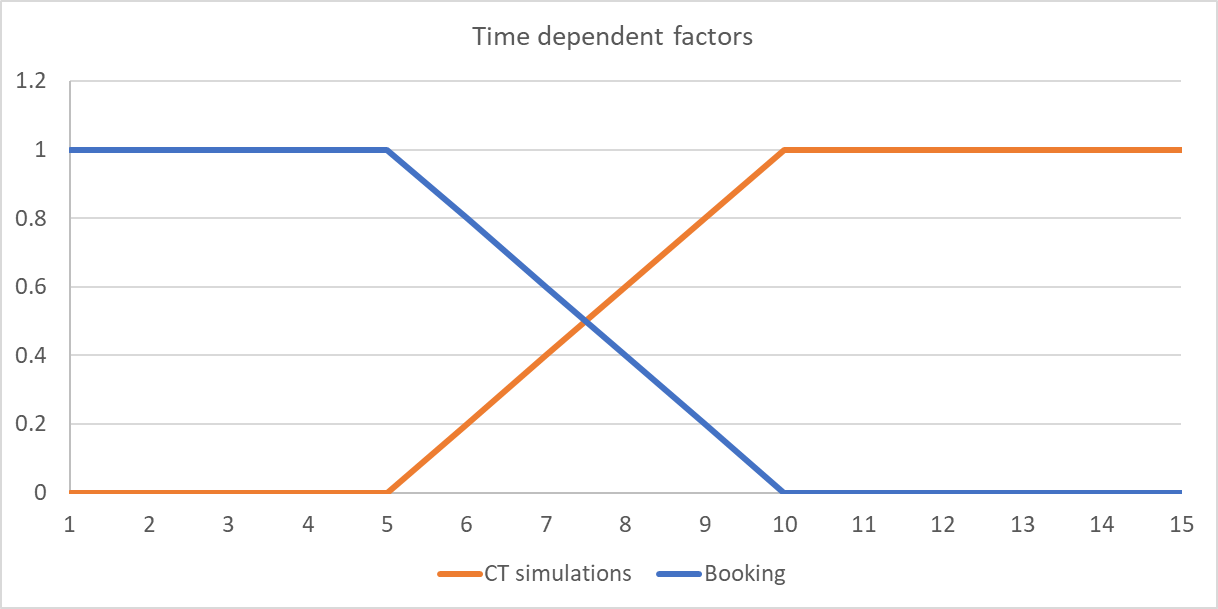


Figure A1. The values of the time dependent factors for booking rate ratio, t_i,b_, and CT simulation ratio, t_i,s_, from days 1 to 15.

Figure A2. Average number of fractions per new start and average time per fraction during January to September 2020.


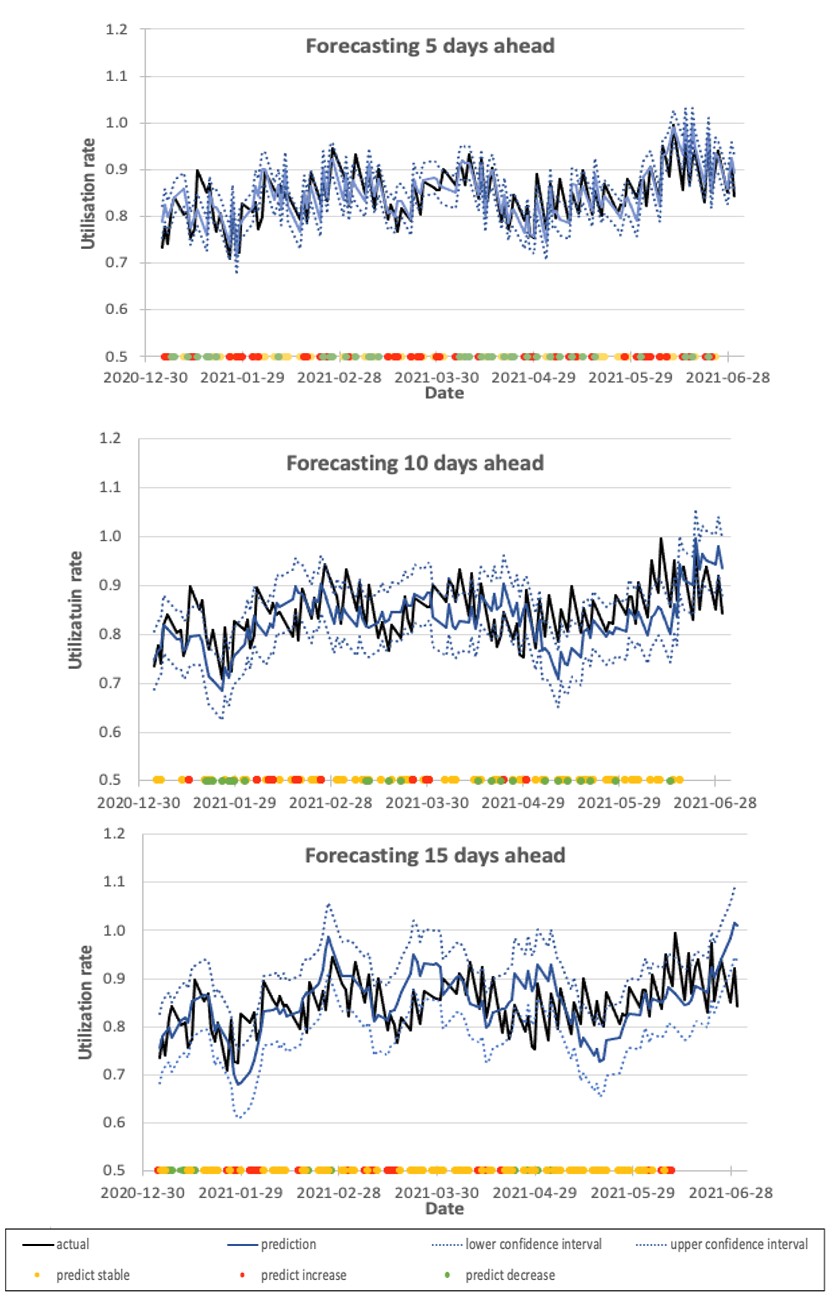


Figure A3. LINAC utilization prediction for day 5, 10, 15 (three curves) during January to June 2021. The colored dots represent the predicted changes, where green indicates predicted decrease, red indicates predicted increase, and yellow indicates predicted stability.
